# Telementored ultrasonography: a narrative review

**DOI:** 10.1590/1516-3180.2020.0607.R2.15092021

**Published:** 2022-03-14

**Authors:** Marcio Luis Duarte, Lucas Ribeiro dos Santos, Wagner Iared, Maria Stella Peccin

**Affiliations:** I MD, MSc. Musculoskeletal Radiologist, WEBIMAGEM, São Paulo (SP), Brazil; and Doctoral Student, Postgraduate Program on Evidence-Based Health, Universidade Federal de São Paulo (UNIFESP), São Paulo (SP), Brazil.; II MD, MSc. Endocrinologist and Professor, Centro Universitário Lusíada (UNILUS), Santos (SP), Brazil; and Doctoral Student, Postgraduate Program on Evidence-Based Health, Universidade Federal de São Paulo (UNIFESP), São Paulo (SP), Brazil.; III MD, PhD. Supervisor Professor, Postgraduate Program on Evidence-Based Health, Universidade Federal de São Paulo (UNIFESP), São Paulo (SP), Brazil.; IV PT, PhD. Associate Professor, Department of Human Movement Sciences, and Advisor, Postgraduate Program on Evidence-Based Health, Universidade Federal de São Paulo (UNIFESP), São Paulo (SP), Brazil.

**Keywords:** Ultrasonography, Teleradiology, Telemedicine, Remote consultation, Teleultrasound, Telesonography, Telementoring

## Abstract

**BACKGROUND:**

Teleradiology consists of electronic transmission of radiological images from one location to another, including between countries, for interpretation and/or consultation. It is one of the most successful applications of telemedicine. Combining this methodology with ultrasound (called telesonography) can accelerate the process of making diagnoses. Despite this rationale, the quality of the evidence about the effectiveness and accuracy of teleradiology remains unknown.

**OBJECTIVE:**

To review the literature on the evidence that exists regarding use of telemedicine for ultrasound in situations of synchronous transmission.

**DESIGN AND SETTING:**

Narrative review conducted within the evidence-based health program at a federal university in São Paulo (SP), Brazil.

**METHODS:**

A search of the literature was carried out in April 2020, in the online databases MEDLINE, EMBASE, Cochrane Library, Tripdatabase, CINAHL and LILACS, for original publications in all languages. The reference lists of the studies included and the main reviews on the subject were also evaluated.

**RESULTS:**

We included ten studies that assessed procedures performed by different healthcare professionals, always with a doctor experienced in ultrasound as a distant mentor. Among these, only one study assessed disease diagnoses in relation to real patients.

**CONCLUSIONS:**

Despite the promising position of telesonography within telemedicine, no studies with reasonable methodological quality have yet been conducted to demonstrate its effectiveness.

## INTRODUCTION

Ultrasonography (US) is a portable and sensitive noninvasive method that does not use ionizing radiation and which is easily available in places where traditional imaging methods are expensive or unavailable.^1,2,3,4,5^

High levels of individual skill in US are difficult to achieve, as the only reference for location and orientation of the image plane for the organ under investigation is the image of this organ on the monitor. There is no positioning feedback to show the examiner how far an image plane deviates from the true axial or cross-sectional plane of a target organ.^6^ Consequently, even experienced examiners may make mistakes regarding image plane positioning.^6^

More and more medical schools, mainly in Europe, have incorporated US training in their curriculum, especially in the discipline of medical emergencies.^7–9^ US requires not only theoretical and anatomical knowledge (which can be acquired through distant-learning methods) but also visual, sensory and motor perceptual skills, along with the ability to integrate ultrasound findings into real-time clinical contexts, as part of decision-making processes.^7,8^

Telemedicine consists of use of telecommunications technologies to communicate and facilitate health-related services between two remote parties. It is typically used in healthcare between provider and patient or between two healthcare providers.^5,10,11,12^ It can serve as a vehicle to enable provision of high-quality, cost-effective, convenient and efficient healthcare for patients while providing access to healthcare services from virtually any location.^5,10,13,14^

Telemedicine can improve the quality of care provided, through complementing the services available or providing care that otherwise would not be available because of time, distance or resource limitations.^10,13,15^ This forms a potential solution for scarcities of medical resources in remote regions.^16,17,18,19^ Low-cost telemedicine technologies can enable rural doctors to access expert support, remote procedure guidance and real-time training opportunities, which may result in reduced transportation costs and improved patient outcomes.^2,13,19,20,21^ The specialties that use it most often are the following:^16,18,22,23,24,25^

- Anesthesiology.
- Cardiology.
- Dermatology.
- Emergency.
- Internal medicine.
- Neurology.
- Obstetrics.
- Pathology.
- Radiology.

Teleradiology consists of electronic transmission of radiological images from one location to another, including between countries, for interpretation and/or consultation. It is one of the most successful and well-structured applications of telemedicine. It has the following objectives:^22,24,25^

- To provide radiological consultations for medical services that do not have local radiological support.
- To facilitate radiological interpretations in on-call situations.
- To provide availability of evaluation and interpretation of radiological images in emergency and non-emergency situations.
- To provide consultative and interpretive radiology services.
- To provide supervision of remote imaging studies.
- To provide support for radiological subspecialties.
- To improve radiological education.
- To promote efficiency and improve the quality of reports.

The only radiologists who need to be on-site are the practical subspecialists for ultrasound and angiography and the interventional radiologist.^22^ Other radiologists can perform their work remotely.^22^ The importance of this becomes clear when it is considered that two-thirds of the world’s population does not have access to imaging examinations.^26^

Tele-ultrasonography, also known as remotely supported ultrasound, teleultrasound, telesonography or telementoring, combines use of ultrasound with telemedicine, thereby allowing interpretation by external specialists. The telesonography process can be conducted in two ways:^26,27^

1. With synchronous transmission, in which the examiner and the specialist are linked through a real-time connection.
2. With asynchronous transmission, in which the images are acquired by an ultrasound operator and later transmitted to a specialist for review.

The advantage of using teleultrasound is that the process of making diagnoses is accelerated. In the case of elective examinations in places with difficult access, use of teleultrasound hastens patients’ return to the primary medical facility, thus improving the continuity of their treatment.^26,28^

## OBJECTIVES

The aim of this study was to review the literature on the evidence that exists regarding use of telemedicine to perform US in situations of synchronous transmission.

## METHODS

For this review, a search of the literature, for original publications, was carried out in April 2020. The following online databases were investigated: Medical Literature Analysis and Retrieval System Online (MEDLINE), Excerpta Medica Database (EMBASE), Cochrane Library, Tripdatabase, Cumulative Index to Nursing and Allied Health Literature (CINAHL) and Latin American and Caribbean Health Sciences Literature (Literatura Latino-Americana e do Caribe em Ciências da Saúde, LILACS). The following Medical Subject Headings (MeSH) terms were used: ultrasonography; teleradiology; telemedicine; remote consultation. The reference lists of studies included and those of the main reviews on the subject were also evaluated. Manual searches were also performed on the reference lists. The full search strategy is presented in Table 1.

**Table 1.** Search strategy according to the corresponding database

Database	Search strategy	Filter	Results
**MEDLINE** (Medical literature analysis and retrieval system online)	#1: “Ultrasonography”[mesh] or (echotomography) or (diagnostic ultrasound) or (diagnostic ultrasounds) or (ultrasound, diagnostic) or (ultrasounds, diagnostic) or (sonography, medical) or (medical sonography) or (ultrasound imaging) or (imaging, ultrasound) or (imagings, ultrasound) or (ultrasound imagings) or (echography) or (ultrasonic imaging) or (imaging, ultrasonic) or (echotomography, computer) or (computer echotomography) or (tomography, ultrasonic) or (ultrasonic tomography) or (diagnosis, ultrasonic) or (diagnoses, ultrasonic) or (ultrasonic diagnoses) or (ultrasonic diagnosis)	Since 2010 Free full text Comparative study Controlled clinical trial Randomized clinical trial Observational study	6,841
	#2: “Teleradiology”[mesh]		
	#3: “Telemedicine”[mesh] or (mobile health) or (health, mobile) or (health) or (telehealth) or (ehealth)		
	#4: “Remote consultation”[mesh] or (consultation, remote) or (teleconsultation) or (teleconsultations)		
	#5: #1 And #2 or #3 or #4		
Cochrane library	#1: “Ultrasonography”[mesh]	Since 2010	2,722
	#2: “Teleradiology”[mesh]		
	#3: “Telemedicine”[mesh]		
	#4: “Remote consultation”[mesh]		
	#5: #1 And #2 or #3 or #4		
**EMBASE** (Excerpta Medica database)	#1: ‘Echography’/exp or ‘diagnostic ultrasonic examination’ or ‘diagnostic ultrasonic imaging’ or ‘diagnostic ultrasonic method’ or ‘diagnostic ultrasound’ or ‘doptone’ or ‘duplex echography’ or ‘echogram’ or ‘echographic evaluation’ or ‘echography’ or ‘echoscopy’ or ‘echosound’ or ‘high resolution echography’ or ‘scanning, ultrasonic’ or ‘sonogram’ or ‘sonographic examination’ or ‘sonographic screening’ or ‘sonography’ or ‘ultrasonic detection’ or ‘ultrasonic diagnosis’ or ‘ultrasonic echo’ or ‘ultrasonic examination’ or ‘ultrasonic scanning’ or ‘ultrasonic scintillation’ or ‘ultrasonogram’ or ‘ultrasonographic examination’ or ‘ultrasonographic screening’ or ‘ultrasonography’ or ‘ultrasound diagnosis’ or ‘ultrasound scanning’	Since 2010 Diagnosis Prospective study Diagnostic test accuracy study Observational study Cohort analysis Cross-sectional study Comparative study Controlled clinical trial	39
	#2: ‘Teleradiology’/exp or ‘tele-radiology’ or ‘teleradiology’		
	#3: ‘Telemedicine’/exp or ‘tele medicine’ or ‘telemedicine’		
	#4: ‘Teleconsultation’/exp or ‘remote consultation’ or ‘tele-consultation’ or ‘teleconsultation’ or ‘telephone consultation’)		
	#5: #1 And #2 or #3 or #4		
**TRIPDATABASE**	#1: “Ultrasonography”	Since 2010	08
	#2: “Teleradiology”		
	#3: “Telemedicine”		
	#4: “Remote consultation”		
	#5: #1 And #2 or #3 or #4		
**CINAHL** (Cumulative index to nursing and allied health literature)	#1: “Ultrasonography”	Since 2010	229
	#2: “Teleradiology”		
	#3: “Telemedicine”		
	#4: “Remote consultation”		
	#5: #1 And #2 or #3 or #4		
**LILACS** (Latin American and Caribbean health sciences literature)	#1: Mh:”ultrassonografia” or (ultrasonografía) or (ultrasonography) or (échographie)or (ecografia) or (ecotomografia computador) or (sonografia médica) or (ecografia médica) or (tomografia ultrassônica) or (diagnóstico ultrassom) or (imagem ultrassônica) or (imagem ultrassonográfica) or (imagem ultrassom) or (imagem ultrassom) or (ecotomografia) or (mh:e01.370.350.850$)	Since 2010 Diagnostic imaging Prospective study Diagnostic study Observational study Controlled clinical trial	04
	#2: Mh:”telerradiologia” or (teleradiology) or (telerradiología) or (téléradiologie) or (mh:e05.920.700$) Or (mh:h02.010.850.700$) Or (mh:h02.403.840.700$) Or (mh:l01.178.847.652.700$) Or (mh:n04.452.515.825.500$) Or (mh:n04.590.374.800.700$) Or (mh:sp2.021.167.010.090.210$) Or (mh:sp2.031.332.210$)		
LILACS (Latin American and Caribbean health sciences literature)	#3: Mh:”telemedicina” or (telemedicine) or (telemedicina) or (télémédecine) or (ciber saúde) or (ciber-saúde) or (cibersaúde) or (disque saúde da mulher) or (medicina 2.0) Or (saúde 2.0) Or (saúde conectada) or (saúde digital) or (saúde eletrônica) or (saúde móvel) or (saúde onipresente) or (saúde pervasiva) or (saúde ubíqua) or (serviço de telemedicina) or (serviço de telessaúde) or (serviços de telemedicina) or (serviços de telessaúde) or (serviços de e-saúde) or (serviços de esaúde) or (serviços em telemedicina) or (tele-serviços em saúde) or (teleassistência) or (telecuidado) or (telecura) or (telessaúde) or (telesserviços de saúde) or (telesserviços em saúde) or (telesserviços na saúde) or (e-saúde) or (esaúde) or (msaúde) or (usaúde) or (mh:h02.403.840$) Or (mh:l01.178.847.652$) Or (mh:n04.590.374.800$) Or (mh:sp2.016.303$) Or (mh:sp2.021.167.010.090$) Or (mh:sp2.031.332$)		
	#4: Mh: ”consulta remota” or (remote consultation) or (consulta remota) or (consultation à distance) or (consulta à distância) or (consultadoria remota) or (consultadoria à distância) or (consultoria remota) or (consultoria à distância) or (teleconsulta) or (teleconsulta assíncrona) or (teleconsulta clínica) or (teleconsulta eletiva) or (teleconsulta síncrona) or (teleconsulta urgente) or (teleconsulta para discussão de casos clínicos) or (teleconsultadoria) or (teleconsultadoria assíncrona) or (teleconsultadoria clínica) or (teleconsultadoria eletiva) or (teleconsultadoria síncrona) or (teleconsultadorias) or (teleconsultas) or (teleconsultoria) or (teleconsultoria assíncrona) or (teleconsultoria clínica) or (teleconsultoria eletiva) or (teleconsultoria síncrona) or (teleconsultoria em urgências) or (teleconsultorias) or (l01.178.847.652.550$) Or (n04.452.758.849.550$) Or (n04.590.374.800.550$) Or (sp2.021.167.010.090.010$) Or (sp2.031.332.010$)		
	#5: #1 And #2 or #3 or #4		

Studies that evaluated teleultrasound in relation to detection of diseases or the quality of ultrasound images, or to make comparisons with the usual ultrasound procedures were included, independent of the study design. All the studies analyzed in this review had an experienced physician as a distant mentor and were published between 2010 and April 2020. There was no language restriction, and no exclusion due to the population size. There was no funding for this study.

## RESULTS

Given the scarcity of good-quality studies on this topic, we included 10 studies that analyzed procedures performed by different healthcare professionals, always with a physician with experience of ultrasound as a distant mentor. Among the studies selected, one was used a prospective cohort;^29^ three were cross-sectional^1,30,31^ (but one of these was non-comparative^30^); four were randomized clinical trials;^2,6,32,33^ one was an observational prospective study;^4^ and one was a prospective randomized crossover study.^34^

Regarding the individuals evaluated, one study evaluated emergency medicine residents with one to two years of previous experience with ultrasound,^4^ and two studies evaluated medical students.^2,6^ The other seven studies, conducted in Canada, South Korea, the United States and Haiti, evaluated non-medical professionals:^1,29–34^ six studies on other healthcare professionals^1,29–31,33,34^ (including sonographers and nurses) and one study on firefighters.^32^ All of these ten studies featured a trained and experienced physician as a distant assessor.

In a randomized clinical trial by Hurst et al.,^33^ 30 sonographers with no previous experience were evaluated. These were divided into three groups: 10 who had had a computer class (two-minute videos); 10 who had had a computer class (two-minute videos) plus telementoring; and 10 who had only had telementoring. The quality of images in Focused Assessment with Sonography in Trauma (FAST*)* examinations was evaluated. In the end, Hurst et al.^33^ concluded that the groups with telementoring obtained better-quality images.

In a prospective randomized study by Smith et al.,^2^ the effectiveness of point-of-care teleultrasound systems consisting of multiple fixed cameras, smartphones and live ultrasound images with real-time audio was evaluated, among 36 medical students from years one to three of their course and among paramedics. The different systems showed similar results, but the smartphones took longer to guide the examination, despite not showing statistical significance. In a second step, the 36 participants were divided into two telementoring groups: one group received telementoring alone, while the other group watched a video before the procedure, which was also carried out with telementoring. These two groups did not present any significant difference regarding their US performance.

Kirkpatrick et al.^32^ conducted a randomized prospective study to evaluate the accuracy of the diagnosis of free liquid in the abdominal cavity, using mannequins, among 101 firefighters with no previous experience with ultrasound, divided into two groups: 53 with telementoring and 48 without telementoring. In the end, the group with telementoring presented diagnostic accuracy of 98.1% while the group without telementoring obtained 95.8%.

In a prospective randomized crossover study by Lee et al.,^34^ 30 novice sonographers were evaluated with regard to assessing the cecal appendix in actors (patients simulating appendicitis), who were divided into two groups: face-to-face and distant mentoring. Among 90 examinations, the appendix was detected within 10 minutes in 82 cases with an onsite expert’s guidance, while in 8 cases it took more than ten minutes of examination to detect the appendix. Among 90 examinations conducted with guidance from a remote expert, 79 appendixes were detected. In 10 cases, the appendix was only detected after more than ten minutes of examination. In one case in the group guided by a remote expert, a failure occurred: the remote expert misinterpreted the terminal ileum as the appendix.

Sheehan et al.^6^ conducted a prospective randomized study in which they evaluated 20 second-year medical students without previous experience with ultrasound. These were divided into two groups that had verbal assistance, but only one received visual guidance (software for expert visual guidance). These authors evaluated the quality of abdominal images and found that the group that received visual guidance produced images of better quality.

In a prospective observational study by Kim et al.,^4^ conducted in South Korea, 12 emergency residents with one to two years of experience with ultrasound were evaluated. These residents performed ultrasound examinations and then, after having completed them, performed the procedure again, this time with telementoring from an experienced doctor. After this procedure, the mentor doctor performed the procedure in person. Residents did not identify the appendix without telementoring, in nine of the 115 ultrasounds performed, but always identified it in the case of appendicitis. With the telementored aid, the appendix was not identified in only two patients. With the onsite analysis, the appendix was identified in all patients. It was concluded that ultrasound with telementoring between an inexperienced doctor and an experienced doctor as a mentor can be used effectively to diagnose acute pediatric appendicitis in an emergency clinical setting, since the results when the specialist provided assistance at a distance were similar to those when the specialist acted in person.

In a cross-sectional study by Levine et al.,^31^ 11 non-medical healthcare workers who had had a theoretical class of 20 minutes and who received assistance at a distance from a doctor while performing ultrasonography were evaluated. The quality of the images was the main outcome. At the end of the study, 91% of the images obtained with the aid of mentoring doctor acting at a distance presented good quality.

Choo et al.^30^ conducted a non-comparative cross-sectional study in which telementoring in echocardiography was evaluated on a simulator, among 33 intensive care nurses without any previous training. With the aid of a mentor, identification of cardiac pathological conditions (anterior myocardial infarction, cardiac tamponade, dilated cardiomyopathy and ventricular fibrillation) became possible in 32 out of 33 examinations.

In a prospective cohort study, Douglas et al.^29^ evaluated 16 non-medical healthcare professionals who had had previous classes on how ultrasound works: 11 through e-learning and five through in-person classes. These professionals’ comfort in performing ultrasound examinations on the internal jugular vein, apex and base of the lungs, cardiac sub-xiphoid view and urinary bladder was evaluated. The authors concluded that, regardless of the teaching group and with the aid of tele-teaching by a doctor, these non-medical healthcare professionals felt comfortable performing ultrasound.

In a cross-sectional study, Robertson et al.^1^ evaluated the application of telementoring to point-of-care ultrasonography in Haiti. Nine non-doctors performed the procedure while using Facetime. They had had a previous 20-minute training session on basic US techniques. The authors evaluated the quality of the images obtained at five anatomical sites: right internal jugular vein, bilateral lung apices, lung bases, heart (subxiphoid view) and bladder; the distant physician reported that 90% of the images were of adequate quality.

A summary of all the studies is presented in Table 2.

**Table 2.** Summary of the studies analyzed

Study	Study design	Participants	Country	Objective	Results
Choo et al.^30^	Non-comparative cross-sectional.	33 Intensive care nurses with no training.	Canada	To identify cardiac pathological conditions using echocardiography in a simulator.	The mentor was able to identify cardiac pathological conditions in 32 out of 33 examinations.
Douglas et al.^29^	Prospective cohort.	16 Non-medical healthcare professionals who had previously had classes on how ultrasound works.	United States	To analyze the comfort of non-medical healthcare professionals for performing ultrasound examinations on the internal jugular vein, apices and bases of the lungs, cardiac sub-xiphoid view and urinary bladder, on a healthy volunteer.	The professionals, regardless of the teaching group and with the aid of teleteaching by a doctor, felt comfortable performing ultrasound examinations.
Hurst et al.^33^	Randomized clinical trial.	30 Sonographers with no previous experience with ultrasound.	United States	To evaluate the quality of the images in the focused assessment with sonography in trauma examination, in a healthy volunteer.	The groups that received telementoring provided images with better quality.
Kim et al.^4^	Prospective observational.	12 Emergency residents with one to two years of experience with ultrasound.	South Korea	To analyze the diagnosis of acute pediatric appendicitis in patients.	Ultrasound with telementoring between an inexperienced doctor and an experienced doctor as a mentor can be effectively used to diagnose acute pediatric appendicitis in an emergency clinic.
Kirkpatrick et al.^32^	Randomized clinical trial.	101 Firefighters with no previous experience with ultrasound.	Canada	To identify free liquid in the abdominal cavity in mannequins, comparing telementoring and no telementoring.	The group with telementoring presented diagnostic accuracy of 98.1%, While the group without telementoring obtained 95.8%.
Lee et al.^34^	Prospective randomized crossover.	30 Novice sonographers.	South Korea	To identify the cecal appendix in actors, comparing the guidance from a remote experts with that of an onsite expert.	The difference between the remote expert’s guidance group and the onsite expert’s group was not significant.
Levine et al.^31^	Cross-sectional.	11 Non-medical healthcare workers who had had a theoretical class of 20 minutes.	United States	To evaluate the quality of images from bedside ultrasound among patients.	91% Of the images had good quality, as assessed by a doctor at a distance.
Robertson et al.^1^	Cross-sectional.	9 Non-physician healthcare workers with 20 minutes of training on basic ultrasound techniques.	United States and Haiti	To evaluate the quality of the images from point-of-care ultrasound on a volunteer.	The distant physician found that 90% of the images were of adequate quality.
Sheehan et al.^6^	Randomized clinical trial.	20 Second-year medical students without previous experience with ultrasound.	United States	To compare verbal and verbal plus visual guidance in identifying the abdominal aorta and right kidney through ultrasound on patients and normal volunteers.	The group that received visual guidance produced images of better quality.
Smith et al.^2^	Randomized clinical trial.	36 Medical students from the first three years of their course and paramedics. All of them were inexperienced in ultrasound.	Canada	To compare different types of telemedicine in ultrasound examinations on the right upper quadrant, on a volunteer.	The different types of telemedicine showed similar results. The group that watched the video did the examination faster than did the group that just received mentoring.

## DISCUSSION

Out of the ten studies analyzed, only one4 actually analyzed diagnoses of diseases in patients with the advent of telementoring in ultrasound. It was concluded that mentoring by an experienced physician improved resident physicians’ capacity to diagnose appendicitis. Choo et al.^30^ and Kirkpatrick et al.^32^ analyzed telementoring among non-medical professionals on a simulator, and obtained favorable results through telementoring, while Douglas et al.^29^ noticed that non-medical healthcare professionals were comfortable with mentoring received from a specialist doctor at a distance. Hurst et al.,^33^ Levine et al.^31^ and Robertson et al.^1^ evaluated the quality of the images produced by non-medical healthcare workers and found sufficient quality for diagnostic evaluation by a specialist doctor. Lee et al.^34^ compared face-to-face mentoring and distant mentoring with regard to defining the location of the cecal appendix, and showed that there was no difference between these types of mentoring. Sheehan et al.^6^ concluded that visual aids for mentoring presented results that were superior to those from telementoring alone. Smith et al.^2^ analyzed different types of telementoring and concluded that there was no difference in their effectiveness.

Jensen et al.^35^ reported that there was good acceptance of telemonitoring for emergency use among medical residents, in relation to point-of-care ultrasonography, despite recognizing a slight improvement in the ultrasound technique over the course of the four months of study. This finding was shared by the mentor doctors, who had more than five years of experience in ultrasound.

Due to advances in technology, innovative techniques can be applied to help perform ultrasound scans, especially in relation to critically ill patients.^36,37,38,39^ These advances include wireless image transmission in the emergency department, and corresponding software that allows remote review and delivery of written feedback from ultrasound specialists via email.^36,37,40,41^

Integration of telemedicine with ultrasonography allows individuals who have no experience with ultrasound to obtain and transmit US images to specialists, i.e. trained and experienced professionals who are located remotely, for them to provide interpretations, especially with regard to targeted and point-of-care assessments.^2,29–31,33,42,43^ Point-of-care ultrasound is a valuable tool that improves the diagnostic potential regarding management of critical patients, especially in resource-constrained environments.^1,35,44^ This situation has already occurred not only with regard to healthcare workers being instructed at a distance (doctors with no experience in ultrasound, nurses and students in the field of healthcare); but also among non-healthcare workers, such as astronauts on the space station, in order to diagnose thrombosis of the internal jugular vein.^31,45^

However, there are barriers to the application of this technique. Distant instructors usually complain about the background noise at the examination location. To solve this problem, some of the alternatives possible include:^46^

- Minimizing the number of people in the examination room.
- Use of rooms with better sound insulation.
- Use of headphones.

Jensen et al.^35^ pointed out that there is a need for a good internet connection and equipment, both for capturing the images and for receiving the images, in order to prevent delays in image transmission (especially in movement, as in cardiac evaluations). This is also needed for receiving the distant physician’s instructions. Lack of a good internet connection would hinder real-time image assessment, which is an essential feature in emergencies.

Another observation mentioned by the resident physicians in the present study was in relation to the limitations on communication between the mentor-physician and the patient, considering that the mentor is in contact with the resident physician through a headset. Nonetheless, however, both the distant physician and the resident physicians preferred the headset to the speakerphone. Added to this, there was difficulty in communication, mainly reported by the distant physician, in relation to limitations on the total movement of the probe device in the resident’s hand, in order to obtain the proper image, which was considered by the residents to be an advantage.

In a systematic review by Marsh-Feiley et al., which showed that a significant proportion of the studies evaluated presented low methodological quality, telesonography was assessed as viable for use in emergency medicine. It has also been shown to have diagnostic power comparable to conventional face-to-face ultrasound. Although the potential benefits have been described, particularly in the context of low-resource environments, they have not been adequately demonstrated in practice, and more robust evidence is required before implementation.^27^ This conclusion, from that review, was similar to what was found in a systematic review by Britton et al., in which low-quality evidence suggested that ultrasound images acquired in environments with limited resources and transmitted using a telemedicine platform to a specialist interpreter had satisfactory quality and value for diagnosis and treatment.^26^

It is noteworthy that in both of the two existing systematic reviews on telementoring in ultrasonography, the studies included presented low methodological quality. Moreover, in all of those studies, there was always a specialist doctor at a distance, as a mentor. For this reason, there is a lack of comparative “head-to-head” studies, in relation to the traditional method of performing ultrasound with telementoring.

### Use of smartphones

It has been speculated that use of new telemedicine and telepresence technologies could potentially eliminate the need for specialists to attend patients face-to-face, thus evincing the usefulness of these technologies for isolated regions and developing countries.^2,37,46^ Most of the benefits from use of smartphones that have been reported have come from very “visual” specialties, in which this tool for easy iconographic communication is probably more relevant than for “less visual” medical specialties.^2,47^ It should nevertheless be taken into account that, today, smartphones are practically omnipresent.^2,48^

Some apps, like WhatsApp, provides immediate interaction among educators and learners, thus providing opportunities to discuss clinical cases and take part in the management performed by residents.^47^ Clavier et al. hypothesized that WhatsApp could have an impact equivalent to that of the practice of exchange groups in clinical thinking.^47^ Likewise, FaceTime can also be applied for teaching and tele-teaching,^1,16^ including for invasive procedures using ultrasound, such as anesthesia.^16^

### Cost savings

Rural physicians often need to travel to the main centers to obtain medical knowledge and practice.^37,49^ In a reverse manner, trained physicians from those main centers need to go to teach in remote regions.^2,37,50^ Both practices are time-consuming and expensive.^2,37,50^ Telementoring offers an economical option for remote environments or for locations with limited resources.^1,2,37,49^

## CONCLUSION

Although telesonography, whether done in “real-time” or not, shows promise within the field of telemedicine, through bringing specialists with a high degree of expertise to remote regions, and assisting in obtaining images and diagnosis, there are still no studies of reasonable methodological quality for demonstrating its efficiency.

Studies with greater methodological rigor, and preferably large-scale randomized clinical trials that evaluate the diagnostic accuracy of the methodology, using multiple telementoring alternatives, along with easily accessible tools and comparisons with the traditional methodology for the procedure, are still necessary in order to reach definitive conclusions.
